# *In vivo* Evaluation of a Newly Synthesized Acetylcholinesterase Inhibitor in a Transgenic *Drosophila* Model of Alzheimer’s Disease

**DOI:** 10.3389/fnins.2021.691222

**Published:** 2021-06-30

**Authors:** Giuseppe Uras, Alessia Manca, Pengfei Zhang, Zsuzsa Markus, Natalie Mack, Stephanie Allen, Marco Bo, Shengtao Xu, Jinyi Xu, Marios Georgiou, Zheying Zhu

**Affiliations:** ^1^Division of Molecular Therapeutics and Formulation, School of Pharmacy, The University of Nottingham, University Park, Nottingham, United Kingdom; ^2^Department of Biomedical Sciences, University of Sassari, Sassari, Italy; ^3^State Key Laboratory of Natural Medicines, Department of Medicinal Chemistry, China Pharmaceutical University, Nanjing, China; ^4^Queens Medical Centre, School of Life Sciences, The University of Nottingham, Nottingham, United Kingdom; ^5^School of Biosciences, University of Nottingham, Nottingham, United Kingdom

**Keywords:** Alzheimer’s disease, Aβ42, acetylcholinesterase, acetylcholinesterase inhibitors, *Drosophila melanogaster*, amyloid aggregation 3

## Abstract

Alzheimer’s disease is a neurodegenerative disease characterized by disrupted memory, learning functions, reduced life expectancy, and locomotor dysfunction, as a result of the accumulation and aggregation of amyloid peptides that cause neuronal damage in neuronal circuits. In the current study, we exploited a transgenic *Drosophila melanogaster* line, expressing amyloid-β peptides to investigate the efficacy of a newly synthesized acetylcholinesterase inhibitor, named XJP-1, as a potential AD therapy. Behavioral assays and confocal microscopy were used to characterize the drug effect on AD symptomatology and amyloid peptide deposition. The symptomatology induced in this particular transgenic model recapitulates the scenario observed in human AD patients, showing a shortened lifespan and reduced locomotor functions, along with a significant accumulation of amyloid plaques in the brain. XJP-1 treatment resulted in a significant improvement of AD symptoms and a reduction of amyloid plaques by diminishing the amyloid aggregation rate. In comparison with clinically effective AD drugs, our results demonstrated that XJP-1 has similar effects on AD symptomatology, but at 10 times lower drug concentration than donepezil. It also showed an earlier beneficial effect on the reduction of amyloid plaques at 10 days after drug treatment, as observed for donepezil at 20 days, while the other drugs tested have no such effect. As a novel and potent AChE inhibitor, our study demonstrates that inhibition of the enzyme AChE by XJP-1 treatment improves the amyloid-induced symptomatology in *Drosophila*, by reducing the number of amyloid plaques within the fruit fly CNS. Thus, compound XJP-1 has the therapeutic potential to be further investigated for the treatment of AD.

## Introduction

Alzheimer’s disease (AD) is recognized as the worldwide leading cause of dementia, with 5.8 million patients currently affected in the US, with this number expected to double by 2050 (Alzheimer’s Association, 2019). Alzheimer’s disease is a progressive neurodegenerative disease with sufferers presenting an array of characteristic phenotypes including amyloid plaques, neurofibrillary tangles, oxidative stress, neuroinflammation, and reduction of cholinergic marker levels, which lead to neuronal death and brain atrophy (Hampel et al., 2018). Amyloid plaques are formed of amyloid-β peptides, secreted by neurons, which form insoluble toxic aggregates that lead to local neuroinflammatory and neurodegenerative responses (Sharma et al., 2019). The amyloidogenic pathway can generate amyloid-β peptides of various lengths, ranging from 17 to 42 amino acids (Wang et al., 2015). Sequential cleavages of the amyloid precursor protein (APP) performed by β- and γ-secretase is required to generate neurotoxic amyloid-β peptides, which is primarily 40 or 42 amino acids in length (O’Brien and Wong, 2011). On the other hand, neurofibrillary tangles are composed of a number of fused Tau protein, due to the presence of a hyperphosphorylated form of Tau (Zhang et al., 2016a). In normal physiological conditions, Tau is required to stabilize and promote microtubule polymerization, while in AD, Tau is hyperphosphorylated, leading to a disruption of microtubule architecture and stability (Sharma et al., 2019).

To date, the AD triggering mechanism is not fully understood, with the exception of genetic mutations causing familial AD where the E693G mutation (hereafter referred to as Arctic mutation) of the APP protein is one of the most severe, due to the rapid aggregation of mutant amyloid-β peptides in the brain (Nilsberth et al., 2001; Norlin et al., 2012; Mendez, 2017). However, according to the cholinergic hypothesis, a drop in the level of cholinergic markers, acetylcholine and acetyl-transferase, is the starting point of AD pathogenesis in the brain (Ramos-Rodriguez et al., 2013; Hampel et al., 2018). Acetylcholine plays a crucial role in memory and learning circuits, which undergo a severe neurodegeneration during AD progression (Mesulam, 2013; Whitehouse et al., 1981). Furthermore, depletion of acetylcholine is linked with an aberrant vasomotor control of the blood–brain barrier (hereafter referred to as BBB), leading to a defective clearance of the amyloid peptides (Hunter et al., 2012).

The Food and Drug Administration (FDA) has currently approved three acetylcholinesterase (AChE) inhibitors for AD treatment; these therapies increase the acetylcholine levels in an attempt to improve the patient’s cognitive functions (Ramos-Rodriguez et al., 2013; Shrivastava et al., 2019). Therapy with AChE inhibitors has clinically resulted in modest disease-modifying effects and beneficial effects in the psychiatric symptomatology (Tayeb et al., 2012). Such therapies also delayed by at least 1 year nursing home placement of AD patients, resulting in a significant reduction of the economic burden experienced by patients’ families (Wattmo et al., 2011). It has been suggested that AChE enzyme facilitates amyloid aggregation by acting as a nucleation point, while treatment with inhibitors reportedly reduced the aggregation rates and potentially prevented or at least delayed the formation of larger plaques (Shrivastava et al., 2019). Thus, development of novel AChE inhibitors that would be able to increase the level of acetylcholine, and therefore improve the cognitive symptomatology and also reduce the available AChE surface for amyloid interaction and subsequent aggregation, remains a promising therapeutic strategy for AD patients.

The FDA has also approved an *n*-methyl D-aspartate (NMDA) receptor antagonist, named memantine, as an AD therapy. Several reports have highlighted the involvement of the glutamatergic system in AD disease progression (Tayeb et al., 2012). Memantine is currently administrated either alone, or in combination with donepezil, in patients with severe cases of AD, despite some disagreeing reports on the further beneficial effects of this combined therapy (Parsons et al., 2013).

Testing the efficacy of AD drugs is particularly complicated by the complex and not fully understood part of the disease. Several models, such as mice, zebrafish, *Drosophila melanogaster*, and cell culture, have been explored to study both the mechanistic biology behind AD pathogenesis and for drug screening purposes (Drummond and Wisniewski, 2017). However, the progress of new drug discovery and development has been slow due to the limited availability of *in vitro* and *in vivo* models that recapitulate AD pathologies, together with neuronal death. This fundamental limit often results in a failure during clinical trials of potential AD therapies, since the results in pre-clinical studies are not confirmed in AD patients. As a result, since 2003, 100% of drug candidates for AD treatment have failed in clinical trials (Cummings et al., 2019). In recognition of the fact that the AD animal models have only limited predictive value for how drugs behave in AD humans, pathologically and genetically relevant models are therefore required for informing the design of more effective therapies.

The common fruit fly *D. melanogaster* has been explored as an AD model, as first reported in 2005 (Crowther et al., 2005). Fruit fly AD models have been exploited to study both pathways involved in AD pathogenesis and to test several potential AD therapies, such as peptides, radiations, natural-derived compounds, and AChE inhibitors (Luo et al., 1992; Zhang et al., 2016b; Pham et al., 2018; Ali et al., 2019; Hwang et al., 2019; Kizhakke et al., 2019; Miyazaki et al., 2019; Zhong et al., 2019; Ogunsuyi et al., 2020). In addition to this, the models offer the possibility to study some human AD patient features, such as reduced lifespan, locomotor defects, and cognitive impairment. The exploitation of the lifespan assay has been extensively used to define the effects of compounds on the reduced survival time caused by the expression of toxic AD proteins. Moreover, the climbing assay allows one to define any locomotor defects caused by AD gene expression and the possibility to detect amelioration following treatment (Kohlhoff et al., 2011; Nichols et al., 2012; Pham et al., 2018; Ali et al., 2019).

However, it has to be mentioned that the role of acetylcholine in invertebrates has some peculiar differences to that observed in the vertebrate organisms. In the fruit fly, acetylcholine is the primary excitatory neurotransmitter in the CNS, and the primary transmitter of sensory neurons (Lee and O’Dowd, 1999). In addition to this, acetylcholine is not present in the neuromuscular junction (hereafter referred to as NMJ) as it is in the vertebrates. Instead, the NMJ of fruit flies or invertebrates is regulated by glutamate. Despite this difference in the NMJ physiology, acetylcholine in the fruit fly regulates jumping, geotaxis, and motion detection stimuli (Hou et al., 2003; Takemura et al., 2013). The fruit fly AChE folding structure is also similar to the vertebrate enzyme, with variations observed mainly in the peripheral anionic site (PAS) but not in the catalytic active site (CAS) (Wiesner et al., 2007).

*Drosophila melanogaster* offers several advantages as a model organism, such as a well-defined genome, the possibility to study hundreds of animals simultaneously, and low cost of maintenance (Sivanantharajah et al., 2019; Tue et al., 2020). Moreover, the easily accessible *Drosophila* brain allows one to image and quantify amyloid plaque deposition in amyloid transgenic models, along with the possibility to investigate selected areas of the brain involved in cognitive processes, such as the mushroom bodies (Hwang et al., 2019; Zhong et al., 2019).

In the work presented here, we drive the expression of the *APP* gene carrying the Arctic mutation in *D. melanogaster* (hereafter referred to as Aβ_arc_ flies) as previously reported (Sandin et al., 2016). The Arctic mutation was initially genotyped from a Swedish family with a history of early-onset familial AD (Nilsberth et al., 2001), causing the production of toxic amyloid-β peptides (Norlin et al., 2012). The Arctic mutation has been linked to a faster amyloid aggregation rate both *in vitro* and *in vivo* (Dahlgren et al., 2002; Englund et al., 2007). In addition, amyloid-β peptides carrying the Arctic mutation form fibrils whose diameter is directly proportional to neuronal cell death rate (Dahlgren et al., 2002), leading to the early onset of the AD symptoms in human patients, including cognitive disabilities, memory impairment, and locomotor defects (Dahlgren et al., 2002; Basun et al., 2008). Following extensive previous *in vitro* studies, here we present biological evaluation of our previously reported novel AChE inhibitor (Wang et al., 2015), named XJP-1, using the Aβ_arc_ *Drosophila* AD model. XJP-1 is a naturally derived product, with part of the structure derived from a compound isolated in the peel of *Musa sapientum* L., which has shown an excellent inhibitory activity against AChE, along with a high specificity for AChE over butyrylcholinesterase (BuChE) (Wang et al., 2015, 2018). In addition to this, XJP-1 was predicted to have high BBB permeability in docking studies and no toxicity on neurotypic SH-SY5Y cells (Wang et al., 2015, 2018).

Here, we examined whether XJP-1, along with current FDA-approved AD therapies, can improve the symptoms induced by the Arctic mutation in *D. melanogaster*, and reduce amyloid deposition in the adult fly brain at different time points.

## Materials and Methods

### Reagents

AChE inhibitors donepezil, galantamine, and rivastigmine were purchased from Sigma-Aldrich Ltd. NMDAR antagonist memantine was purchased from Sigma-Aldrich Ltd. Drug candidate XJP-1 was gifted by Prof. Jinyi Xu (China Pharmaceutical University).

### Fly Strains

The UAS-APPE693G (#33774) and *elav*-Gal4 (#458) flies were purchased from Bloomington Drosophila Stock Centre Indiana. Flies were kept at 25°C in 25-ml plastic vials containing 5 ml of standard fly food. *elav*-Gal4 virgin females were crossed with UAS-APPE693G males, all stocks were kept at 25°C, and the female progeny was used for all the assays. *Elav-*Gal4 flies were used as a wild-type (WT) control.

### Fly Food Preparation

Flies were fed using the standard fly food recipe containing (quantity for 1 L): 70.62 ml of golden syrup, 15.87 g of yeast, 9.12 g of soya flour, 67 g of cornmeal, 5.25 g of agar, and 711.25 ml of water. To add the drug of interest into the food, an aliquot of the drug stock solution was dissolved in a volume of water to 1/5th of the total food volume and mixed on the magnetic stirrer for 2 min. Subsequently, once the temperature has cooled to <80°C, the diluted drug was added and mixed in homogeneously. Compounds were dissolved at minimum effective concentration, as 40 μM for XJP-1, 0.5 mM for donepezil, 0.5 mM for galantamine, 0.1 mM for rivastigmine, and 0.5 mM for memantine. Combined therapies were administrated at the same concentration as the monotherapy.

### Lifespan Assay

Newly emerged flies aged 0–24 h were collected and placed into fresh food vials containing 5 ml of fly food. Each vial contained a total of 20 flies. At least 100 flies per genotype were analyzed. Food vials containing the flies were kept horizontally and flies were flipped 3 times/week, with the number of dead flies counted at that time. Flies that escaped, died in the food, or became trapped in the cotton bung were not taken into account.

### Climbing Assay

WT and transgenic *Drosophila* locomotor functions were measured by performing a climbing assay. Flies aged 0–24 h were collected and placed into fresh food vials containing the appropriate treatment. Each food vial contained up to 10 flies. Vial containing flies were flipped three times a week, for a total of 3 weeks, and at this time, climbing performance was recorded as follows: flies were moved into an empty 50-ml plastic tube, and the number of flies that had climbed above the three cutoff lines (40, 30, and 20 ml) was counted after 20 s. The results were then calculated using the following formula:

Climbing Index = (percentage of total flies above the 40-ml line) × 1

+ (percentage of total flies between the 30-ml and 40-ml lines) × 0.75

+ (percentage of total flies between the 20-ml and 30-ml lines) × 0.50.

### Immunohistochemistry

Adult flies were firstly anesthetized with CO_2_ and subsequently killed in pure ethanol (100%). Brains were dissected in phosphate buffered saline (PBS) and then fixed in 4% paraformaldehyde (PFA) for 20 min at room temperature (RT). Tissues were then washed three times in 1% PBS-Tween 20 (PBS-T) for 2 min, and subsequently placed in 5% normal goat serum (NGS) in PBS-T for 2 h at room temperature. After removing the 5% NGS-T, the rabbit anti-Aβ42 primary antibody (#ab2539, Abcam plc.) was added at a final concentration of 1:200 in 5% NGS-T and left to incubate overnight at +4°C. The primary antibody was then washed three times with 1% PBS-T and subsequently the goat anti-rabbit secondary antibody was added to a final 1:250 concentration. The sample was allowed to incubate for 1 h at room temperature. Goat anti-rabbit 488 Alexa Fluor secondary antibody (#A-11034; ThermoFisher Scientific) was then washed three times with 1% PBS-T. Brain samples were then mounted for confocal imaging exploiting a “bridge” structure to avoid any sample damage caused by the coverslip. Two 0.1-mm thin coverslips were attached to the glass slide at approximately 5 mm distance to each other. Subsequently, 20 μl of mounting media (90% PBS and 10% glycerol) was added in the gap between the two coverslips. Samples were then placed into the mounting media, and an additional cover slip to bridge the gap between the two “pylon” coverslips. Samples were analyzed by confocal microscopy within 24 h from mounting.

### Confocal Imaging

All brain images were acquired using the Zeiss880 confocal microscope. Laser power was set at 2.5%, gain at 520. Digital offset was set at 200. The imaging acquisition speed was 1.03 s, averaging two times per slice. For visualization purposes, the figures presented have been made by setting the brightness value to 30,000 and subsequently converting into 8-bit images.

### Image Analysis

A macro was created in FIJI to identify and measure amyloid spots in the images scanned with the confocal microscope. The processing analysis was carried out using the following steps: raw 16 bit confocal images were imported into FIJI; maximum projection (focus stack flattening) was applied and a copy of this image was created; the copy was used for object identification and the creation of the mask; a Gaussian blur filter was applied to the image to smooth the noise pixels; a threshold of 14,850–61,890 was used to separate the plaque signals from the rest of the image and from the background; the lower value of thresholding 14,850 was determined by using negative control and non-stained areas of the brain. Top value was set just below the maximum to avoid occasionally appearing large clumps of overexposed areas; a mask was created based on the threshold areas; particle analysis was carried out on the mask while fluorescence intensity measurements were performed by redirecting the measure function to the original image; the particle analysis was carried out to generate size, intensity, and shape measurements of the plaques; the number of particles (amyloid spots) was used to characterize the effect of each treatment.

### Western Blot

A total of 50 fly heads were dissected and frozen overnight at -80°C. Samples were then homogenized in 150 μl of RIPA Lysis and Extraction Buffer (#89901, ThermoFisher Scientific). Subsequently, each sample was centrifuged at 14,000*g* at 4°C for 10 min, and the supernatant was then transferred into a fresh new vial. Protein concentrations were quantified using Bradford Assay Kit (#ab102535, Abcam, United Kingdom). According to protein concentrations, samples were then dissolved in 4 × NuPage LDS Sample Buffer (#NP0007, Invitrogen) and loaded into 4%–12% Bis-Tris Gel (#NP0322PK2, Invitrogen). Following separation, proteins were transferred onto a 0.2-μm nitrocellulose membrane (#1620150, Bio-Rad) using semi-dry apparatus TransBlot Turbo (#1704150, Bio-Rad). Membranes were then blocked in 5% bovine serum albumin (BSA) for 1 h at room temperature and subsequently incubated overnight at 4°C with rabbit anti-Aβ_42_ primary antibody (#ab2539, Abcam plc) at final 1:1000 concentration. Monoclonal Anti-β actin antibody was used as loading control in Western Blot experiments (#ab1801, AbCam plc.). Each membrane was then washed three times in 5% TBS-T for 5 min and subsequently incubated for 1 h at room temperature with goat-anti rabbit IgG H&L (HRP) (#ab6721, AbCam plc.) secondary antibody at a final concentration of 1:5000. Excess secondary antibody was then discarded and membrane was washed three times using 5% TBS-T. Each membrane was then imaged using Fujifilm LAS-4000 (Fujifilm). Results were then analyzed using FIJI.

### Quantification of AChE-Induced Aβ_42_ Aggregation

Measurements of AChE-induced amyloid aggregation were taken following the protocol previously reported (Jiang et al., 2019). Briefly, hexafluoroisopropanol (HFIP)-treated E22G Aβ peptides (#SP-Ab-11_0.1, JPT–Innovative Peptide Solution) were dissolved in DMSO to reach a final 200 μM stock. The dissolved peptides were subsequently centrifuged at 13,500*g* for 10 min, and the supernatant was then transferred into a fresh vial and used for the following experiments. To evaluate the aggregation rate in the presence of AChE inhibitors, 2 μl of the compound of interest (at the appropriate concentration) was added into each vial, followed by 2 μl of 200 μM Aβ peptides stock, 20 μl of AChE (#C3389, Sigma-Aldrich Ltd) (2 U/ml, in 1 × PBS at pH 8.0), and 76 μl 1 × PBS, pH 8.0. The reaction was then incubated at room temperature (RT) for 24 h. Subsequently, 100 μl of 5 μM Thioflavin T (#ab120751, ThT) was added into each vial. After 1-h incubation at RT, fluorescence emission was recorded at 490 nm with an excitation wavelength of 450 nm using a Tecan Spark microplate reader. Results were then processed as previously described by Jiang et al. (Jiang et al., 2019) using the subsequent formula: (F_*i*_ - F_*b*_)/(F_*o*_ - F_*b*_) - 100, where F_*i*_ corresponds to amyloid aggregation in the presence of peptides, AChE, AChE inhibitors, and ThT; F_*o*_ represents the amyloid aggregation in presence of peptides, AChE, and ThT; and F_*b*_ corresponds to blank control containing ThT only.

### Statistical Analysis

All statistical analyses were performed using GraphPad Prism 9 software. Data obtained were firstly tested for normality using the Shapiro–Wilk test. The Kaplan–Meier test was used to compare different survival curves. The Kruskal–Wallis test, followed by Dunn’s *post hoc*, was used to compare differences between three or more groups in non-normally distributed data. The ANOVA test was used to compare differences between three or more groups of normally distributed samples. The Friedman test, followed by Dunn’s *post hoc* test, was used to analyze differences between three or more groups in the climbing assay. Each experiment was performed in triplicate, and all results are presented as mean ± standard error of the mean (SEM) or mean ± standard deviation (SD). Results with a *P*-value < 0.05 were considered significant.

## Results

### XJP-1 Treatment Improves Life Expectancy in Aβ_arc_ Flies

**FIGURE 1:**
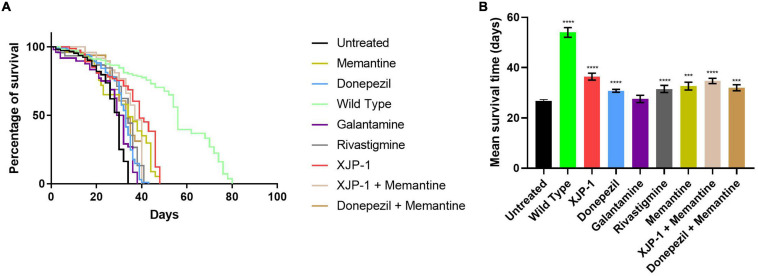
Aβ_arc_ flies’ lifespan. **(A)** Kaplan–Meier survival trajectories of Aβ_Arc_ flies under different drug treatments. **(B)** Mean survival time of Aβ_Arc_ flies on different treatments. Kruskal–Wallis test followed by Dunn’s *post hoc* was used to compare the differences between different groups. Data are expressed as mean ± SEM, *n* = 3 (number of independent experiments with a minimum of 100 flies per genotype). *P* < 0.05 was considered as significant. *P < 0.05; **P < 0.01; ***P < 0.001; ****P < 0.0001. Genotypes: Untreated = Aβ_arc_-expressing flies; Wild Type = *elav-*Gal4 line; all treated groups are Aβ_arc_-expressing flies fed with the stated drug.

In order to investigate the effect of the Aβ_arc_ mutation on life expectancy, flies were firstly grown on food without any therapy. Expression of amyloid peptides resulted in a dramatic drop of mean survival time when compared to WT flies (Figures 1A,B). To investigate the effects of XJP-1 treatment on life expectancy, Aβ_arc_ flies were treated with 40 μM (as the minimum effective concentration) of the new AChE inhibitor after testing a range of concentrations (10–40 μM); 10 to 30 μM did not produce any significant amelioration in pilot lifespan assay (data not shown). XJP-1 therapy resulted in a significant increase in the mean survival time, 26. 74 days vs. 36.38 days (Table 1), of Aβ_arc_ flies with around 40% of the entire population surviving after day 40 (Figures 1A,B). Comparable results were also recorded with other FDA-approved AD drugs, including the AChE inhibitors donepezil (30.76 days) (0.5 mM as the minimum effective concentration), rivastigmine (31.49 days) (0.1 mM), and the NMDAR antagonist memantine (32.61 days) (0.5 mM). Galantamine (0.5 mM), however, had no significant effect (Table 1). Among all the tested compounds, XJP-1 showed most potent efficacy at a concentration of 40 μM, more than 10 times lower than observed for donepezil, on Aβ_arc_ survival time (Figures 1A,B).

**TABLE 1:** Data table.

		**Aβarc**	**XJP-1**		**Donepezil**		**Rivastigmine**		**Galantamine**		**Memantine**		**XJP-1 (Memantine)**		**Donepezil (Memantine)**	
	
		**Mean**	**Mean**	**P value vs. Aβarc**	**Mean**	**P value vs. Aβarc**	**Mean**	***P* value vs. Aβarc**	**Mean**	***P* value vs. Aβarc**	**Mean**	***P* value vs. Aβarc**	**Mean**	***P* value vs. Aβarc**	**Mean**	***P* value vs. Aβarc**
Lifespan assay		26.74	36.38	<0.0001	30.76	<0.0001	31.49	<0.0001	27.52	0.9994	32.61	0.0008	34.68	<0.0001	31.98	0.0005
Climbing assay	Day-2:	35.625	67.81	0.016	87.5	0.0003	87.083	0.0005	67.75	0.0511	67.868	0.041	37	0.932	72.6375	0.032
	Day-5:	34.3329	40.86	0.0724	75	0.006	66.75	0.0003	86.008	0.0088	79.86	0.0005	71.25	0.0088	70.5145	0.0003
	Day-7:	23.6085	50.08	0.04	38.5	0.028	56.223	0.0005	36.174	0.58	59.903	0.0006	68.26	0.0007	65.3487	0.0004
	Day-9:	12.5175	38.278	0.0014	27.1	0.77	31.25	0.042	21.166	0.61	27.487	0.059	36.93	0.0056	29.8625	0.0071
	Day-12:	9.5967	44.4	0.0008	29.5	0.019	24.438	0.58	31.832	0.0532	14.25	0.66	19.68	0.0839	10.685	0.7643
	Day-14:	6.925	31.05	0.022	33.6	0.0047	0	0.865	21.583	0.0799	23.369	0.062	44.35	0.0006	20.7625	0.0664
	Day-16:	3.6242	67.29	0.0002	49.7	0.0003	3.261	0.832	12.5	0.0527	23.369	0.052	11.84	0.054	0.06273	0.71
	Day-19:	4.886	15.418	0.032	18.7	0.012	2	0.75453	5.556	0.93	7.293	0.057	5.313	0.513	0.24573	0.361
	Day-21:	0.832	8.369	0.1734	8.13	0.0615	5.716	0.566	1.667	0.91	0	0.65	0	0.66	0	0.982
Amyloid spots (10 days)		987.5	362.3	0.0005	703.9	0.2691	773.1	0.5659	711.6	0.2961	639	0.1095	453	0.0035	886.3	0.984
Amyloid spots (20 days)		1137	383.5	<0.0001	390.5	<0.0001	525.7	0.0582	673.4	0.0657	373.5	<0.0001	520.2	0.0002	528	0.0096
MB + FBS amyloid spots (10 days)		465.3	196	0.0034	358.8	0.5605	325.4	0.2709	296	0.1204	307.6	0.1686	210.4	0.006	269.4	0.519
MB + FBS amyloid spots (20 days)		324.6	154.2	0.0029	133.2	0.0008	191.2	0.27	230.4	0.1934	133.2	0.0008	113.6	0.0002	164.4	0.0055
Medulla + OL (10 days)		459.7	219.2	0.0675	431	0.9996	308.4	0.2195	270.2	0.4465	285.2	0.2961	216.8	0.0635	331.8	0.6279
Medulla (OL (20 days)		607	321.2	0.0126	304.2	0.0075	419.4	0.1776	175	0.0014	253.8	<0.0001	202.4	0.0003	225	0.0006
Amyloid aggregation		93.85	23.52	0.0001	24.6	0.0002	56.12	0.0354	44.61	0.005	N/A	N/A	26.72	0.0002	33.72	0.0008

*Comparison of mean and *P-*values of different treatment groups against the untreated Aβ_arc_ flies.*

Since the FDA currently approves a combined therapy of memantine and donepezil, we investigated whether XJP-1 and memantine treatment would further enhance the results obtained by XJP-1 alone. Both a combination of FDA-approved therapies and XJP-1 plus memantine showed a significant improvement of the Aβ_arc_ *Drosophila* lifespan (Figures 1A,B). However, no significant difference was recorded when the combined therapies were compared to the corresponding monotherapies (Table 1).

### Locomotor Functions Improve Following XJP-1 Treatment

**FIGURE 2:**
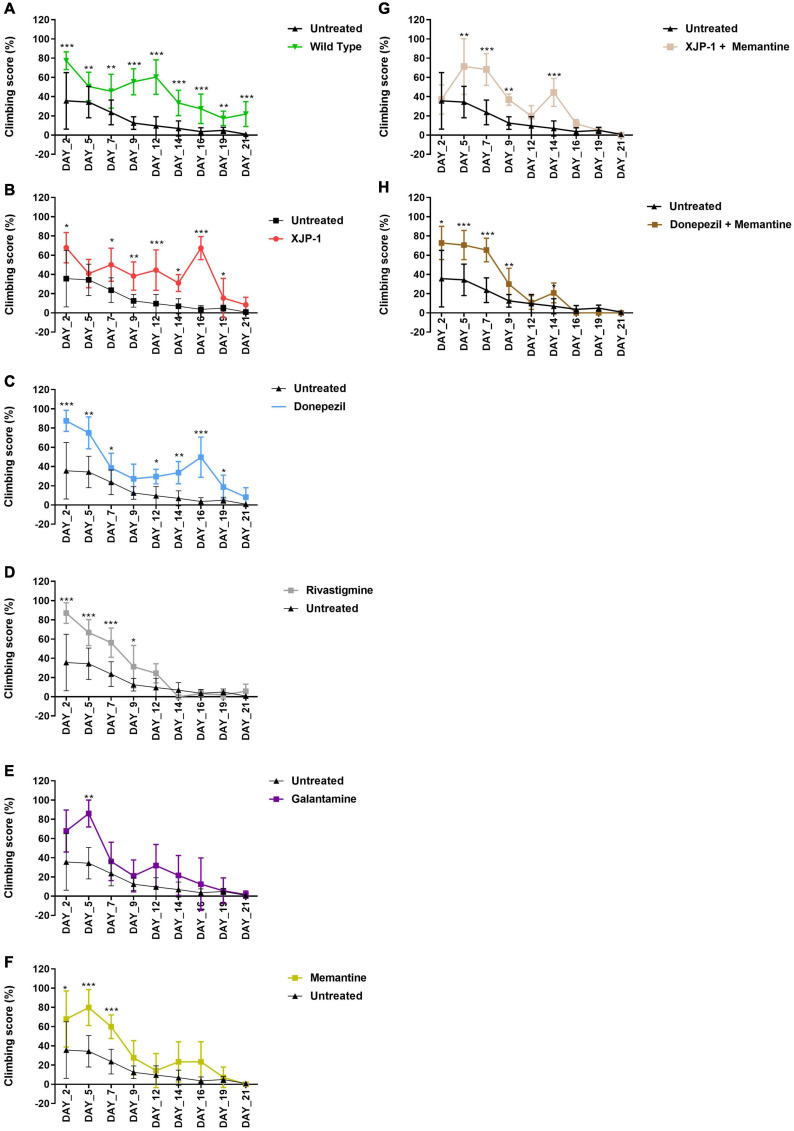
Aβ_arc_ flies climbing assay. Aβ_arc_ flies climbing assay under different treatments. Data show the climbing performance trends over a 21-day period. Data generated from the climbing index was processed as a percentage of the total. In order to compare vials with a different number of flies, repeated measures analysis of variance was used to compare climbing scores between treated and untreated groups. **(A)** Climbing assay of untreated Aβ_arc_ flies. **(B)** Climbing assay of Aβ_arc_ flies treated with XJP-1. **(C)** Climbing assay of Aβ_arc_ flies treated with donepezil. **(D)** Climbing assay of Aβ_arc_ flies treated with rivastigmine. **(E)** Climbing assay of Aβ_arc_ flies treated with galantamine. **(F)** Climbing assay of Aβ_arc_ flies treated with memantine. **(G)** Climbing assay of Aβ_arc_ flies treated with XJP-1 and memantine. **(H)** Climbing assay of Aβ_arc_ flies treated with donepezil and memantine. Data are presented in the figure as the mean ± SD; data are compared against the untreated group. *n* = 3 (number of independent experiments, each experiment with a minimum of 10 flies per treatment). *P* < 0.05 was considered as significant. *P < 0.05; **P < 0.01; ***P < 0.001; ****P < 0.0001.

The climbing assay is a behavioral test, based on negative geotaxis against gravity, which is used to assess the locomotor function in *Drosophila* (Nichols et al., 2012). With this assay, Aβ_arc_ flies recorded a time-dependent worsening of climbing ability, thereby faithfully recapitulating symptoms observed in human patients (Figure 2A). Treatment with XJP-1 improved locomotor function in Aβ_arc_ flies over 20 days, with the exception of days 5 and 21 of analysis (Figure 2B). The climbing performance recorded in flies treated with XJP-1 were constantly twofold better than the untreated Aβ_arc_ flies, with peaks at day 16 of analysis when the treated group recorded a climbing score of 67.29 against 3.62 for the untreated groups (Table 1).

On the other hand, among the FDA-approved therapies, only donepezil treatment showed results comparable to XJP-1, recording a homogeneous climbing performance over the first 19 days of analysis, with a minimum of twofold improvement of the locomotor performance, except for day 9 of analysis (Figure 2C; Table 1).

Rivastigmine, galantamine, and memantine had a beneficial effect that was limited only to the first 10 days of analysis (Figures 2D–F). Aβ_arc_ flies treated with combined therapies showed a significant improvement in locomotor function only throughout the first 10 days of analysis, and at day 14 for XJP-1 and memantine, therefore having a worse effect on locomotor functions than donepezil and XJP-1 alone (Figures 2G,H, Table 1).

### XJP-1 Reduces the Number of Amyloid Plaques in the Brain

**FIGURE 3:**
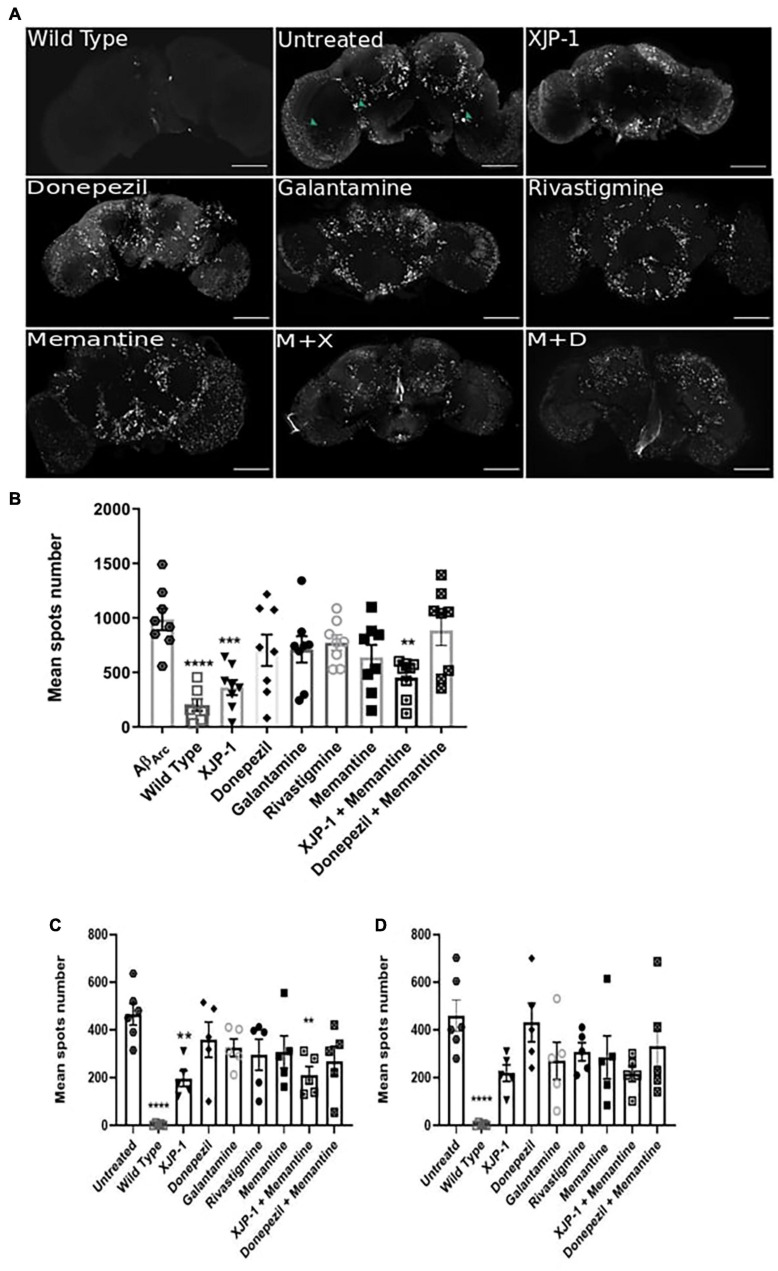
Amelioration of amyloid spots in Aβ_arc_ flies CNS after 10 days of treatment. **(A)** Representative confocal images of WT (top left panel) or Aβ_arc_ brains; arrows show amyloid spots; scale bar: 100 μm. **(B)** Whole brain quantification of amyloid spots. ANOVA test followed by Bonferroni’s *post hoc* was used to compare the differences between three or more groups, *P* < 0.05 was considered as significant. Data are presented as mean ± SEM. *n* = 3, with a minimum of five flies per experiment analyzed. *P < 0.05; **P < 0.01; ***P < 0.001; ****P < 0.0001. **(C)** Mushroom body and fan-shaped body quantification of amyloid spots. ANOVA test followed by Bonferroni’s *post hoc* was used to compare the differences between three or more groups, *P* < 0.05 was considered as significant. Data are presented as mean ± SEM. *n* = 3, with a minimum of five flies per experiment analyzed. *P < 0.05; **P < 0.01; ***P < 0.001; ****P < 0.0001. **(D)** Medulla and Optic lobe quantification of amyloid spots. ANOVA test followed by Bonferroni’s *post hoc* was used to compare the differences between three or more groups, *P* < 0.05 was considered as significant. Data are presented as mean ± SEM. *n* = 3, with a minimum of five flies per experiment analyzed. *P < 0.05; **P < 0.01; ***P < 0.001; ****P < 0.0001.

The deposition, accumulation, and aggregation of amyloid peptides in the central nervous system (CNS) is a crucial point in the development of Alzheimer’s symptomatology. Using HFIP-treated Aβ peptides, together with confocal microscopy, we found Aβ_arc_ fly brains to possess large and extensively distributed amyloid aggregates (hereafter referred to as amyloid spots) at 10 days post-eclosion (Figure 3A). To investigate whether flies treated with XJP-1, or clinically available therapies, would present any difference in amyloid pathology in the CNS, we imaged Aβ_arc_ fly brains following 10 days of treatment (Figure 3A). Subsequent quantification showed a significant reduction in amyloid spot counts in flies treated with either XJP-1 or XJP-1 and memantine combined. None of the clinically approved therapies had similar results to the new AChE inhibitor (Figure 3B). To further investigate whether there was a remarkable difference in amyloid spot counts in those brain areas rich in cholinergic neurons, we exploited the Fly Brain Observatory software to determine areas of the brain that are rich in cholinergic neurons. We found that mushroom bodies (Supplementary Figure 1A), fan-shaped bodies (Supplementary Figure 1B), the medulla (Supplementary Figure 1C), and optic lobes (Supplementary Figure 1D), were rich in cholinergic neurons compared to other areas, such as the antennal lobes (Supplementary Figure 1E) and suboesophageal ganglion (Supplementary Figure 1F) (Xu et al., 2020). Analysis of mushroom body and fan-shaped body areas recorded a significant reduction of amyloid spots in flies treated with both XJP-1 monotherapy and XJP-1 and memantine combined therapy (Figure 3C). Conversely, none of the treatments tested had a beneficial impact on the medulla and optic lobe amyloid spot count (Figure 3D).

**FIGURE 4:**
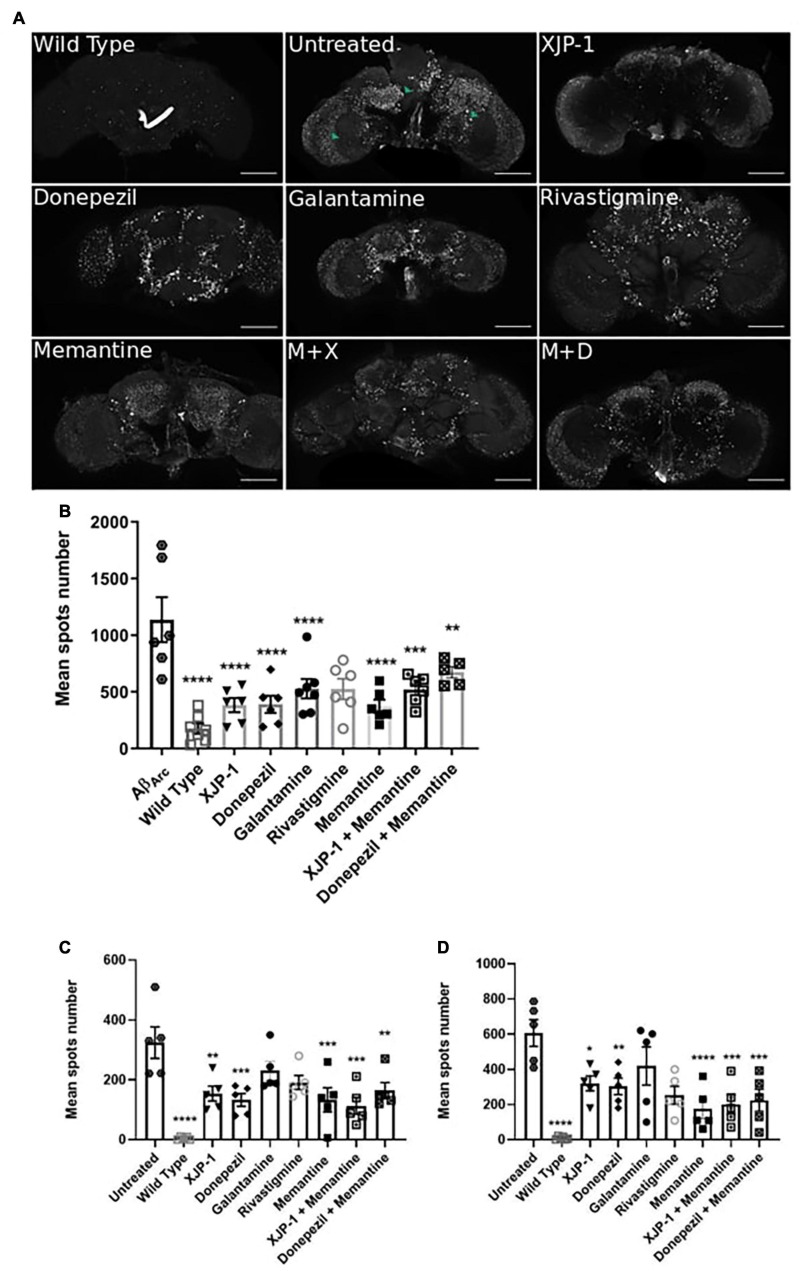
Amelioration of amyloid spots in Aβ_arc_ flies CNS after 20 days of treatment. **(A)** Representative confocal images of WT (top left panel) or Aβ_arc_ brains; arrows show amyloid spots; scale bar: 100 μm. **(B)** Whole brain quantification of amyloid spots. ANOVA test followed by Bonferroni’s *post hoc* was used to compare the differences between three or more groups, *P* < 0.05 was considered as significant. Data are presented as mean ± SEM. *n* = 3, with a minimum of 1 fly per experiment analyzed. *P < 0.05; **P < 0.01; ***P < 0.001; ****P < 0.0001. **(C)** Mushroom body and fan-shaped body quantification of amyloid spots. ANOVA test followed by Bonferroni’s *post hoc* was used to compare the differences between three or more groups, *P* < 0.05 was considered as significant. Data are presented as mean ± SEM. *n* = 3, with a minimum of 1 fly per experiment analyzed. *P < 0.05; **P < 0.01; ***P < 0.001; ****P < 0.0001. **(D)** Medulla and Optic lobe quantification of amyloid spots. ANOVA test followed by Bonferroni’s *post hoc* was used to compare the differences between three or more groups, *P* < 0.05 was considered as significant. Data are presented as mean ± SEM. *n* = 3, with a minimum of five flies per experiment analyzed. *P < 0.05; **P < 0.01; ***P < 0.001; ****P < 0.0001.

To assess whether AD progression alters these amyloid spot count results, we also analyzed the Aβ_arc_ fly brains 20 days post-eclosion (Figure 4A). The whole brain analysis showed a significant reduction of the amyloid spot counts, similar to that observed with XJP-1 at 10 days, in flies treated with XJP-1, donepezil, and memantine, while galantamine and rivastigmine failed to show any significant effect (Figure 4B). The same reduction trends were recorded in the mushroom bodies and fan-shaped bodies (Figure 4C). Although none of the treatments tested reduced the amyloid spot counts in the medulla and optical lobe areas after 10 days (Figure 3D), analysis of samples treated for 20 days showed a significant decrease in the amyloid spot levels for all treatment tested, with the exception of galantamine and rivastigmine (Figure 4D). The combined therapies investigated, however, did not show any further beneficial effects than the single therapies (Table 1).

### XJP-1 Reduces Amyloid Aggregation *via* AChE Inhibition

**FIGURE 5:**
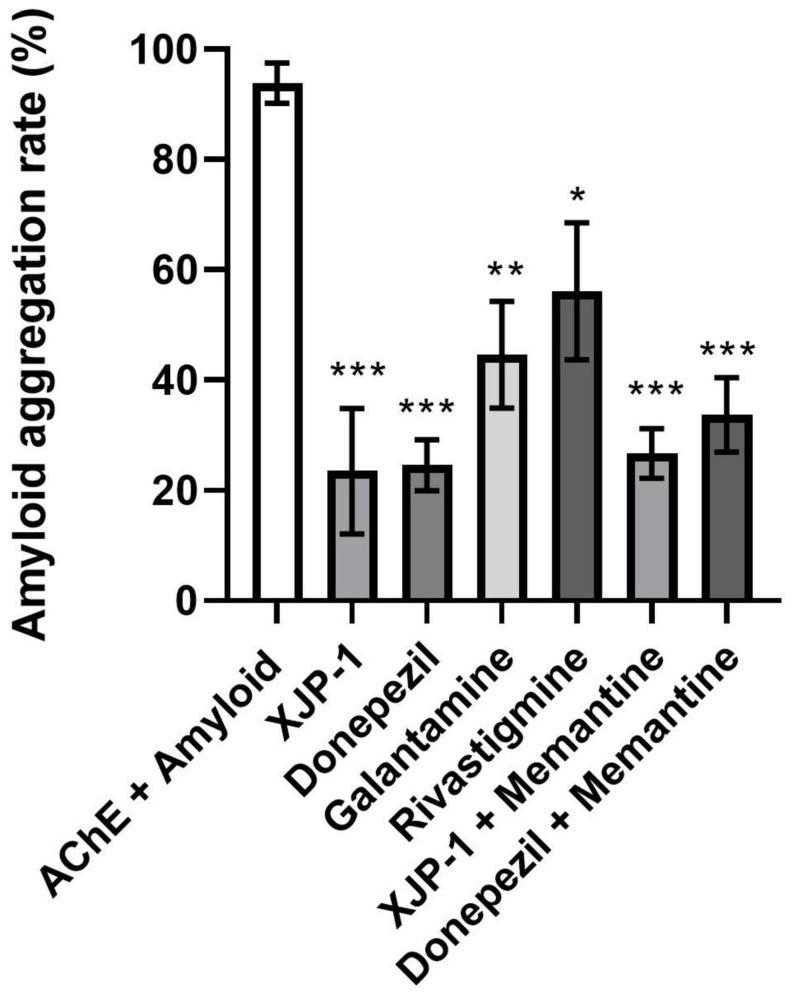
Reduction of AChE-induced Aβ-peptide aggregation rates in the presence of AChE inhibitors. ANOVA test followed by Bonferroni’s *post hoc* was used to compare the differences between three or more groups. Data are presented as mean ± SEM of *n* = 3. *P < 0.05; **P < 0.01; ***P < 0.001; ****P < 0.0001.

To confirm that the observed reduction in amyloid spots was not a result of a reduced quantity of amyloid peptides within the Aβ_arc_ fly brains, we quantified amyloid peptide expression following 10 and 20 days of treatment. At both time points, and for all drug treatments, amyloid peptide expression was statistically similar to the untreated brains (Supplementary Figure 2). This result is expected since the expression of the transgene is determined by the UAS/Gal4 system and is not targeted by any of the drugs studied. We hypothesized that the AChE enzyme was functioning as a nucleation point for amyloid peptide aggregation, as previously reported (Inestrosa et al., 1996; Rees et al., 2003; Lushchekina et al., 2017). We therefore used an *in vitro* enzymatic assay to evaluate amyloid peptide aggregation rate, where amyloid-β peptides carrying the Arctic mutation were co-incubated with AChE enzyme in the presence, or not, of all the AChE inhibitors tested. We found a significant drop in all treatments studied, with the greatest reductions observed for XJP-1 and donepezil, both recording an almost 80% decrease in aggregation rate (Figure 5). Once again, the combined therapies did not result in any further decrease in amyloid aggregation rate (Figure 5).

## Discussion

Over the past 10 years, the fruit fly has emerged as a powerful *in vivo* model for neurodegenerative diseases, including AD (Zhang et al., 2016a; Pham et al., 2018; Ali et al., 2019; Cornelison et al., 2019; Higham et al., 2019; Hwang et al., 2019; Miyazaki et al., 2019; Sivanantharajah et al., 2019; Tue et al., 2020). Transgenic *Drosophila* AD models have been employed for a number of drug testing studies involving different targets such as AChE, GSK-3β, lysozyme, radiation, Tau protein, and dopaminergic receptors (Sandin et al., 2016; Zhang et al., 2016a; Pham et al., 2018; Ali et al., 2019; Hwang et al., 2019; Miyazaki et al., 2019; Zhong et al., 2019). Flies use a variety of neurotransmitters to communicate between neurons (e.g., dopamine, GABA, glutamate, acetylcholine, serotonin); however, differences have been observed in the functions of these neurotransmitters between invertebrates and vertebrates. In Drosophila, glutamate is known to be the primary excitatory neurotransmitter at the NMJ, whereas acetylcholine plays this role in mammals (Jan and Jan, 1976; Colombo and Francolini, 2019). Conversely, in the mammalian CNS, glutamate is the primary excitatory neurotransmitter. In flies, acetylcholine is broadly expressed and is the primary excitatory neurotransmitter in the CNS, while the role of the glutamatergic system in the Drosophila brain remains rather ambiguous (Buchner, 1991). In fact, there is evidence that glutamate can be inhibitory within certain fly CNS systems, such as in the olfactory and visual systems (Liu and Wilson, 2013; Molina-Obando et al., 2019). Although differences in the functions of neurotransmitter systems should be taken into account when interpreting results, if using the fly to model neurodegenerative diseases, it should be noted that it is the damaged cholinergic portion of the brain that is linked to the development of Alzheimer’s disease, hence the use of cholinesterase inhibitors to slow disease development. The ability to use Drosophila as a model for human neurodegenerative diseases has proven to be, and will continue to be, a powerful tool to understand and suppress degenerative mechanisms (Lenz et al., 2013).

In this work, we have demonstrated the capacity of a new AChE-I compound, XJP-1, to ameliorate amyloid-β deposition and consequent behavioral phenotypes in the Aβ_arc_ transgenic *Drosophila* AD model. Treatment with XJP-1 resulted in an increased survival time of Aβ_arc_ flies of more than 10 days, improving as well the locomotor functions of the transgenic fruit flies. Similar results were scored by the FDA therapy donepezil, despite being administrated at a concentration 10-fold higher than XJP-1, in contrast with previous reports of donepezil being effective at a concentration as low as 30 μM (Chakraborty et al., 2011; Pham et al., 2018).

XJP-1 was able to reduce the amyloid spot counts as early as 10 days after commencing the treatment, in crucial areas of the *Drosophila* brain for memory and learning functions. Among the clinically available AChE inhibitors, none of them showed such an early effect on amyloid spot counts, with only donepezil having similar results as XJP-1, but after 20 days of administration and at a concentration more than 10 times higher. This is the first evidence that XJP-1 therapy results in an overall improvement of AD symptomatology *in vivo*.

On the other hand, a combined treatment of XJP-1 and memantine did not result in further significant improvement when compared to XJP-1 treatment alone, as well as combined donepezil and memantine therapy. Combined therapy is usually only given to patients when they enter an advanced stage of the disease, when memantine is added to patients already on donepezil therapy (Tayeb et al., 2012; Parsons et al., 2013). In our study, all drugs were administrated continuously from 24 h upon eclosion, including combined therapies, giving a possible explanation on why donepezil with memantine, and XJP-1 with memantine, did not show any further beneficial effect when compared to single therapy on this AD model. This limitation may also explain the weaker efficacy of galantamine and rivastigmine compared to donepezil, as they are currently prescribed to Alzheimer’s patients within mild to moderate stages of AD, while donepezil is given to AD patients at all stages of the disease, from mild to severe (Haake et al., 2020). Moreover, the NMDAR antagonist memantine is also administered during all stages of AD, alone or as combination with donepezil to further improve the cognitive functions, as efficacy against the amyloid pathology is still controversial (Folch et al., 2018).

Over the past decade, the efforts to find a cure to AD have been mainly focused on anti-amyloid agents, such as beta-secretase 1 (BACE1) inhibitors, assuming that the aggregation of amyloid-β peptides into plaques is the central triggering mechanism. However, a number of clinical trials involving this type of target have failed to produce any significant improvement (Cummings et al., 2019). Thus, it is necessary to investigate novel therapeutics for which the mechanism of action involves directly, or indirectly, multiple targets such as the cholinergic pathway, amyloid pathology, and Tau hyperphosphorylation. A number of studies have placed the cholinergic system at the center of the AD etiopathogenesis, involving the overactivation of crucial enzymes, such as BACE1 and GSK3-β (Noh et al., 2009; Yoshiyama et al., 2010; Medeiros et al., 2011; Potter et al., 2011; Kalkman and Feuerbach, 2016; Zhang et al., 2016a). Our study has shown a clear involvement of AChE inhibition on amyloid aggregation resulting in an amelioration of symptoms. The effect of XJP-1 on Aβ_arc_ transgenic flies were partially replicated only by donepezil at a concentration 10 times higher than our drug candidate, with XJP-1 having an earlier beneficial effect in reducing the amyloid spot counts after 10 days of treatment. Other than that, the other FDA-approved AChE inhibitors, galantamine and rivastigmine, reduced the amyloid aggregation rate of about 50%, while XJP-1 and donepezil reduced it by about 80%, potentially explaining the limited beneficial effects observed in Aβ_arc_ flies treated with galantamine and rivastigmine. In addition to this, docking studies carried out on XJP-1 showed a dual-binding property for both the PAS and CAS of the AChE enzyme (Wang et al., 2015), which may further justify the low dosage needed to slow down the amyloid aggregation, since the PAS of AChE has been linked to increased amyloid-β peptide aggregation rate (Inestrosa et al., 1996). Despite not being tested in this study, XJP-1 has been predicted to have anti-inflammatory and anti-oxidant properties due to its natural product-derived structure, which may have also played a role in ameliorating the AD symptomatology in Aβ_arc_ flies (Wang et al., 2015, 2018).

Taken together, the results of these experiments suggest that XJP-1 ameliorates AD symptomatology by indirectly reducing amyloid aggregation *via* AChE inhibition. Despite XJP-1 showing promising efficacy with this AD model, further studies will be necessary to investigate whether XJP-1 results can be translated into mammalian models and whether treatment can also improve Tau-related phenotypes presented by AD patients.

## Conclusion

This work demonstrates that XJP-1 is an effective and potent AD drug candidate, which efficiently rescues AD symptoms in an Aβ_arc_ fly model, with earlier effects at a much lower concentration than observed for any of the currently approved therapies. This includes a significant improvement in locomotor defects and lifespan. By inhibiting AChE, XJP-1 indirectly reduced amyloid aggregation and therefore also the number of amyloid spots detected at an earlier stage in Aβ_arc_ *Drosophila* brains.

## Data Availability Statement

The raw data supporting the conclusions of this article will be made available by the authors, without undue reservation.

## Author Contributions

GU and AM equally contributed to this work. GU conceived the study and its experimental design, contributed to samples collection, carried out the samples analysis, analyzed the results, performed research, and wrote the manuscript. AM and ZM contributed to sample collection and carried out the sample analysis. MB reviewed the manuscript. PZ, SX, and JX synthesized and supplied XJP-1 and reviewed the manuscript. MG, SA, NM, and ZZ conceived the study and its experimental design and reviewed and approved the manuscript. All authors contributed to the article and approved the submitted version.

## Conflict of Interest

The authors declare that the research was conducted in the absence of any commercial or financial relationships that could be construed as a potential conflict of interest.

## Supplementary Material

Supplementary Figure 1 Representative images of cholinergic neurons network in *Drosophila Melanogaster* brain obtained from the Virtual Fly Observatory. **(A)** Cholinergic neurons within the mushrooms bodies. **(B)** Cholinergic neurons within the fan-shaped bodies. **(C)** Cholinergic neurons within the medulla. **(D)** Cholinergic neurons within the optic lobes. **(E)** Cholinergic neurons within the antennal lobes. **(F)** Cholinergic neurons within the subesophageal ganglion.

Supplementary Figure 2 Amyloid peptides quantity in Aβ_arc_ flies’ heads. **(A)** Representative images of WB membranes showing amyloid peptides quantity after 10 and 20 days of treatment. **(B)** Aβ_Arc_ peptide quantification by membrane image analysis. ANOVA test followed by Bonferroni’s *post hoc* was used to compare the differences between different groups. Data are presented as mean ± SEM, *n* = 3 (number of independent experiments with 50 fly heads per treatment group). *P* < 0.05 was considered as significant. ^∗^*P* < 0.05; ^∗∗^*P* < 0.001; ^∗∗∗^*P* < 0.0001.

## Abbreviations

AChE: Acetylcholinesterase
AD: Alzheimer’s disease
APP: amyloid precursor protein
A β _arc_: amyloid- β Arctic
BACE1: β -site APP-cleaving enzyme 1
BuChE: butyrylcholinesterase
BSA: bovine serum albumin
BBB: blood–brain barrier
CAS: catalytic active site
*elav*: embryonic-lethal abnormal visual
FDA: Food and Drug Administration
GSK3- β: Glycogen synthase kinase 3 beta
HFIP: hexafluoroisopropanol
M + D: memantine + donepezil
M + X: memantine + XJP-1
NGS: normal goat serum
NMJ: neuromuscular junction
NMDA: *n*-methyl D-aspartate
NMDAR: *n*-methyl D-aspartate receptor
PAS: peripheral anionic site
RIPA: Radioimmunoprecipitation assay buffer
ThT: thioflavin-T
UAS: upstream activating sequence

## References

[B1] Ali F. Rahul, Jyoti S. Naz F. Ashafaq M. Shahid M. (2019). Therapeutic potential of luteolin in transgenic *Drosophila* model of Alzheimer’s disease. *Neurosci. Lett.* 692 90–99. 10.1016/j.neulet.2018.10.053 30420334

[B2] Alzheimer’s Association (2019). Available online at: www.alz.org

[B3] Basun H. Bogdanovic N. Ingelsson M. Almkvist O. Näslund J. Axelman K. (2008). Clinical and neuropathological features of the arctic app gene mutation causing early-onset Alzheimer disease. *Arch. Neurol.* 65 499–505. 10.1001/archneur.65.4.499 18413473 PMC2723757

[B4] Buchner E. (1991). Genes expressed in the adult brain of drosophila and effects of their mutations on behavior: a survey of transmitter- and second messenger-related genes. *J. Neurogenet.* 7 153–192. 10.3109/01677069109167432 1679453

[B5] Chakraborty R. Vepuri V. Mhatre S. D. Paddock B. E. Miller S. Michelson S. J. (2011). Characterization of a *Drosophila* Alzheimer’s disease model: pharmacological rescue of cognitive defects. *PLoS One* 6:e20799. 10.1371/journal.pone.0020799 21673973 PMC3108982

[B6] Colombo M. N. Francolini M. (2019). Glutamate at the vertebrate neuromuscular junction: from modulation to neurotransmission. *Cells* 8:996. 10.3390/cells8090996 31466388 PMC6770210

[B7] Cornelison G. L. Levy S. A. Jenson T. Frost B. (2019). Tau-induced nuclear envelope invagination causes a toxic accumulation of mRNA in Drosophila. *Aging Cell* 18:e12847. 10.1111/acel.12847 30411463 PMC6351838

[B8] Crowther D. C. Kinghorn K. J. Miranda E. Page R. Curry J. A. Duthie F. A. (2005). Intraneuronal Abeta, non-amyloid aggregates and neurodegeneration in a *Drosophila* model of Alzheimer’s disease. *Neuroscience* 132 123–135. 10.1016/j.neuroscience.2004.12.025 15780472

[B9] Cummings J. Lee G. Ritter A. Sabbagh M. Zhong K. (2019). Alzheimer’s disease drug development pipeline: 2019. *Alzheimer’s Dementia* 5 272–293. 10.1016/j.trci.2019.05.008 PMC6617248 31334330

[B10] Dahlgren K. N. Manelli A. M. Stine W. B. Jr. Baker L. K. Krafft G. A. LaDu M. J. (2002). Oligomeric and fibrillar species of amyloid-beta peptides differentially affect neuronal viability. *J. Biol. Chem.* 277 32046–32053. 10.1074/jbc.m201750200 12058030

[B11] Drummond E. Wisniewski T. (2017). Alzheimer’s disease: experimental models and reality. *Acta Neuropathol.* 133 155–175. 28025715 10.1007/s00401-016-1662-x PMC5253109

[B12] Englund H. Sehlin D. Johansson A. S. Nilsson L. N. Gellerfors P. Paulie S. (2007). Sensitive ELISA detection of amyloid-beta protofibrils in biological samples. *J. Neurochem.* 103 334–345. 17623042 10.1111/j.1471-4159.2007.04759.x

[B13] Folch J. Busquets O. Ettcheto M. Sánchez-López E. Castro-Torres R. D. Verdaguer E. (2018). Memantine for the treatment of dementia: a review on its current and future applications. *J. Alzheimer’s Dis. JAD.* 62 1223–1240. 10.3233/jad-170672 29254093 PMC5870028

[B14] Haake A. Nguyen K. Friedman L. Chakkamparambil B. Grossberg G. T. (2020). An update on the utility and safety of cholinesterase inhibitors for the treatment of Alzheimer’s disease. *Expert Opin. Drug Saf.* 19 147–157. 10.1080/14740338.2020.1721456 31976781

[B15] Hampel H. Mesulam M. M. Cuello A. C. Farlow M. R. Giacobini E. Grossberg G. T. (2018). The cholinergic system in the pathophysiology and treatment of Alzheimer’s disease. *Brain* 141 1917–1933. 29850777 10.1093/brain/awy132 PMC6022632

[B16] Higham J. P. Levy S. A. Jenson T. Frost B. (2019). Alzheimer’s disease associated genes ankyrin and tau cause shortened lifespan and memory loss in *Drosophila*. *Front. Cell. Neurosci.* 13:260. 10.3389/fncel.2019.00260 31244615 PMC6581016

[B17] Hou D. Suzuki K. Wolfgang W. J. Clay C. Forte M. Kidokoro Y. (2003). Presynaptic impairment of synaptic transmission in *Drosophila* embryos lacking Gs(alpha). *J. Neurosci.* 23 5897–5905. 10.1523/jneurosci.23-13-05897.2003 12843294 PMC6741247

[B18] Hunter J. M. Kwan J. Malek-Ahmadi M. Maarouf C. L. Kokjohn T. A. Belden C. (2012). Morphological and pathological evolution of the brain microcirculation in aging and Alzheimer’s disease. *PLoS One* 7:e36893. 10.1371/journal.pone.0036893 22615835 PMC3353981

[B19] Hwang S. Jeong H. Hong E. H. Joo H. M. Cho K. S. Nam S. Y. (2019). Low-dose ionizing radiation alleviates Abeta42-induced cell death via regulating AKT and p38 pathways in *Drosophila* Alzheimer’s disease models. *Biol. Open.* 8:bio036657. 10.1242/bio.036657 PMC6398453 30670376

[B20] Inestrosa N. C. Alvarez A. Perez C. A. Moreno R. D. Vicente M. Linker C. (1996). Acetylcholinesterase accelerates assembly of amyloid-beta-peptides into Alzheimer’s fibrils: possible role of the peripheral site of the enzyme. *Neuron* 16 881–891. 10.1016/s0896-6273(00)80108-7 8608006

[B21] Jan L. Y. Jan Y. N. (1976). L-glutamate as an excitatory transmitter at the *Drosophila* larval neuromuscular junction. *J. Physiol.* 262 215–236. 10.1113/jphysiol.1976.sp011593 186587 PMC1307638

[B22] Jiang C. S. Ge Y. X. Cheng Z. Q. Song J. L. Wang Y. Y. Zhu K. (2019). Discovery of new multifunctional selective acetylcholinesterase inhibitors: structure-based virtual screening and biological evaluation. *J. Comput. Aided Mol. Des.* 33 521–530. 10.1007/s10822-019-00202-2 30989573

[B23] Kalkman H. O. Feuerbach D. (2016). Modulatory effects of alpha7 nAChRs on the immune system and its relevance for CNS disorders. *Cell Mol. Life. Sci.* 73 2511–2530. 10.1007/s00018-016-2175-4 26979166 PMC4894934

[B24] Kizhakke P. A. Olakkaran S. Antony A. Tilagul K. S. Hunasanahally P. G. (2019). Convolvulus pluricaulis (Shankhapushpi) ameliorates human microtubule-associated protein tau (hMAPτ) induced neurotoxicity in Alzheimer’s disease *Drosophila* model. *J. Chem. Neuroanat.* 95 115–122. 10.1016/j.jchemneu.2017.10.002 29051039

[B25] Kohlhoff K. J. Jahn T. R. Lomas D. A. Dobson C. M. Crowther D. C. Vendruscolo M. (2011). The iFly tracking system for an automated locomotor and behavioural analysis of *Drosophila melanogaster*. *Integr. Biol. (Camb)* 3 755–760. 10.1039/c0ib00149j 21698336 PMC5011414

[B26] Lee D. O’Dowd D. K. (1999). Fast excitatory synaptic transmission mediated by nicotinic acetylcholine receptors in *Drosophila* neurons. *J. Neurosci.* 19 5311–5321. 10.1523/jneurosci.19-13-05311.1999 10377342 PMC6782340

[B27] Lenz S. Karsten P. Schulz J. B. Voigt A. (2013). Drosophila as a screening tool to study human neurodegenerative diseases. *J. Neurochem.* 127 453–460. 10.1111/jnc.12446 24028575

[B28] Liu W. W. Wilson R. I. (2013). Glutamate is an inhibitory neurotransmitter in the *Drosophila* olfactory system. *Proc. Natl. Acad. Sci. U S A.* 2013:201220560. 10.1073/pnas.1220560110 PMC3690841 23729809

[B29] Luo L. Tully T. White K. (1992). Human amyloid precursor protein ameliorates behavioral deficit of flies deleted for Appl gene. *Neuron* 9 595–605. 10.1016/0896-6273(92)90024-8 1389179

[B30] Lushchekina S. V. Kots, Novichkova D. A. Petrov K. A. Masson P. (2017). Role of acetylcholinesterase in β-Amyloid aggregation studied by accelerated molecular dynamics. *BioNanoScience* 7 396–402. 10.1007/s12668-016-0375-x

[B31] Medeiros R. Kitazawa M. Caccamo A. Baglietto-Vargas D. Estrada-Hernandez T. Cribbs D. H. (2011). Loss of muscarinic M1 receptor exacerbates Alzheimer’s disease-like pathology and cognitive decline. *Am. J. Pathol.* 179 980–991. 10.1016/j.ajpath.2011.04.041 21704011 PMC3157199

[B32] Mendez M. F. (2017). Early-Onset Alzheimer disease. *Neurol. Clin.* 35 263–281. 10.1016/j.ncl.2017.01.005 28410659 PMC5407192

[B33] Mesulam M. M. (2013). Cholinergic circuitry of the human nucleus basalis and its fate in Alzheimer’s disease. *J. Comp. Neurol.* 521 4124–4144. 10.1002/cne.23415 23852922 PMC4175400

[B34] Miyazaki H. Okamoto Y. Motoi A. Watanabe T. Katayama S. Kawahara S. I. (2019). Adzuki bean (Vigna angularis) extract reduces amyloid-beta aggregation and delays cognitive impairment in Drosophila models of Alzheimer’s disease. *Nutr. Res. Pract.* 13 64–69. 10.4162/nrp.2019.13.1.64 30788058 PMC6369114

[B35] Molina-Obando S. Vargas-Fique J. F. Henning M. Gür B. Schladt T. M. Akhtar J. (2019). ON selectivity in the *Drosophila* visual system is a multisynaptic process involving both glutamatergic and GABAergic inhibition. *eLife* 8:e49373. 10.7554/eLife.49373 PMC6845231 31535971

[B36] Nichols C. D. Becnel J. Pandey U. B. (2012). Methods to assay *Drosophila* behavior. *JVE* 61:e3795. 10.3791/3795 22433384 PMC3671839

[B37] Nilsberth C. Westlind-Danielsson A. Eckman C. B. Condron M. M. Axelman K. Forsell C. (2001). The ‘Arctic’ APP mutation (E693G) causes Alzheimer’s disease by enhanced Abeta protofibril formation. *Nat. Neurosci.* 4 887–893. 10.1038/nn0901-887 11528419

[B38] Noh M. Y. Koh S. H. Kim Y. Kim H. Y. Cho G. W. Kim S. H. (2009). Neuroprotective effects of donepezil through inhibition of GSK-3 activity in amyloid-beta-induced neuronal cell death. *J. Neurochem.* 108 1116–1125. 10.1111/j.1471-4159.2008.05837.x 19077054

[B39] Norlin N. Hellberg M. Filippov A. Sousa A. A. Grobner G. Leapman R. D. (2012). Aggregation and fibril morphology of the Arctic mutation of Alzheimer’s Abeta peptide by CD. TEM, STEM and in situ AFM. *J. Struct. Biol.* 180 174–189. 10.1016/j.jsb.2012.06.010 22750418 PMC3466396

[B40] O’Brien R. J. Wong P. C. (2011). Amyloid precursor protein processing and Alzheimer’s disease. *Annu. Rev. Neurosci.* 34 185–204. 21456963 10.1146/annurev-neuro-061010-113613 PMC3174086

[B41] Ogunsuyi O. B. Oboh G. Oluokun O. O. Ademiluyi A. O. Ogunruku O. O. (2020). Gallic acid protects against neurochemical alterations in transgenic *Drosophila* model of Alzheimer’s disease. *Adv. Traditional Med.* 20 89–98. 10.1007/s13596-019-00393-x

[B42] Parsons C. G. Danysz W. Dekundy A. Pulte I. (2013). Memantine and cholinesterase inhibitors: complementary mechanisms in the treatment of Alzheimer’s disease. *Neurotox. Res.* 24 358–369. 10.1007/s12640-013-9398-z 23657927 PMC3753463

[B43] Pham H. M. Xu A. Schriner S. E. Sevrioukov E. A. Jafari M. (2018). Cinnamaldehyde improves lifespan and healthspan in *Drosophila melanogaster* models for Alzheimer’s disease. *Biomed. Res. Int.* 2018:3570830. 10.1155/2018/3570830 PMC6136480 30228985

[B44] Potter P. E. Kitazawa M. Caccamo A. Baglietto-Vargas D. Estrada-Hernandez T. Cribbs D. H. (2011). Pre- and post-synaptic cortical cholinergic deficits are proportional to amyloid plaque presence and density at preclinical stages of Alzheimer’s disease. *Acta Neuropathol.* 122 49–60. 10.1007/s00401-011-0831-1 21533854 PMC3362487

[B45] Ramos-Rodriguez J. J. Pacheco-Herrero M. Thyssen D. Murillo-Carretero M. I. Berrocoso E. Spires-Jones T. L. (2013). Rapid beta-amyloid deposition and cognitive impairment after cholinergic denervation in APP/PS1 mice. *J. Neuropathol. Exp. Neurol.* 72 272–285. 10.1097/nen.0b013e318288a8dd 23481704 PMC3612835

[B46] Rees T. Hammond P. I. Soreq H. Younkin S. Brimijoin S. (2003). Acetylcholinesterase promotes beta-amyloid plaques in cerebral cortex. *Neurobiol. Aging* 24 777–787. 10.1016/s0197-4580(02)00230-0 12927760

[B47] Sandin L. Bergkvist L. Nath S. Kielkopf C. Janefjord C. Helmfors L. (2016). Beneficial effects of increased lysozyme levels in Alzheimer’s disease modelled in *Drosophila melanogaster*. *FEBS J.* 283 3508–3522. 10.1111/febs.13830 27562772 PMC5132093

[B48] Sharma P. Srivastava P. Seth A. Tripathi P. N. Banerjee A. G. Shrivastava S. K. (2019). Comprehensive review of mechanisms of pathogenesis involved in Alzheimer’s disease and potential therapeutic strategies. *Prog. Neurobiol.* 174 53–89. 10.1016/j.pneurobio.2018.12.006 30599179

[B49] Shrivastava S. K. Sinha S. K. Srivastava P. Tripathi P. N. Sharma P. Tripathi M. K. (2019). Design and development of novel p-aminobenzoic acid derivatives as potential cholinesterase inhibitors for the treatment of Alzheimer’s disease. *Bioorg. Chem.* 82 211–223. 10.1016/j.bioorg.2018.10.009 30326403

[B50] Sivanantharajah L. Mudher A. Shepherd D. (2019). An evaluation of *Drosophila* as a model system for studying tauopathies such as Alzheimer’s disease. *J. Neurosci. Methods* 319 77–88. 10.1016/j.jneumeth.2019.01.001 30633936

[B51] Takemura S.-Y. Bharioke A. Lu Z. Nern A. Vitaladevuni S. Rivlin P. K. (2013). A visual motion detection circuit suggested by *Drosophila* connectomics. *Nature* 500 175–181. 10.1038/nature12450 23925240 PMC3799980

[B52] Tayeb H. O. Yang H. D. Price B. H. Tarazi F. I. (2012). Pharmacotherapies for Alzheimer’s disease: beyond cholinesterase inhibitors. *Pharmacol. Ther.* 134 8–25. 10.1016/j.pharmthera.2011.12.002 22198801

[B53] Tue N. T. Dat T. Q. Ly L. L. Anh V. D. Yoshida H. (2020). Insights from Drosophila melanogaster model of Alzheimer’s disease. *Front. Biosci. (Landmark Ed)* 25:134–146. 10.2741/4798 31585881

[B54] Wang C. Wu Z. Cai H. Xu S. Liu J. Jiang J. (2015). Design, synthesis, biological evaluation and docking study of 4-isochromanone hybrids bearing N-benzyl pyridinium moiety as dual binding site acetylcholinesterase inhibitors. *Bioorg. Med. Chem. Lett.* 25 5212–5216. 10.1016/j.bmcl.2015.09.063 26454504

[B55] Wang J. Wang C. Wu Z. Li X. Xu S. Liu J. (2018). Design, synthesis, biological evaluation, and docking study of 4-isochromanone hybrids bearing N-benzyl pyridinium moiety as dual binding site acetylcholinesterase inhibitors (part II). *Chem. Biol. Drug Des.* 91 756–762. 10.1111/cbdd.13136 29112799

[B56] Wattmo C. Wallin A. K. Londos E. Minthon L. (2011). Risk factors for nursing home placement in Alzheimer’s disease: a longitudinal study of cognition, ADL, service utilization, and cholinesterase inhibitor treatment. *Gerontologist* 51 17–27. 10.1093/geront/gnq050 20562471

[B57] Whitehouse P. J. Price D. L. Clark A. W. Coyle J. T. DeLong M. R. (1981). Alzheimer disease: evidence for selective loss of cholinergic neurons in the nucleus basalis. *Ann. Neurol.* 10 122–126. 10.1002/ana.410100203 7283399

[B58] Wiesner J. Kriz Z. Kuca K. Jun D. Koca J. (2007). Acetylcholinesterases – the structural similarities and differences. *J. Enzyme. Inhib. Med. Chem.* 22 417–424. 10.1080/14756360701421294 17847707

[B59] Xu C. S. Januszewski M. Lu Z. Takemura S.-Y. Hayworth K. J. Huang G. (2020). A connectome of the adult *Drosophila* central brain. *bioRxiv [preprint]* 10.1101/2020.01.21.911859 PMC7546738 32880371

[B60] Yoshiyama Y. Kojima A. Ishikawa C. Arai K. (2010). Anti-inflammatory action of donepezil ameliorates tau pathology, synaptic loss, and neurodegeneration in a tauopathy mouse model. *J. Alzheimers. Dis.* 22 295–306. 10.3233/jad-2010-100681 20847440

[B61] Zhang B. Li Q. Chu X. Sun S. Chen S. (2016a). Salidroside reduces tau hyperphosphorylation via up-regulating GSK-3beta phosphorylation in a tau transgenic *Drosophila* model of Alzheimer’s disease. *Transl. Neurodegener.* 5:21. 10.1186/s40035-016-0068-y PMC5126879 27933142

[B62] Zhang B. Wang Y. Li H. Xiong R. Zhao Z. Chu X. (2016b). Neuroprotective effects of salidroside through PI3K/Akt pathway activation in Alzheimer’s disease models. *Drug Des. Dev. Ther.* 10 1335–1343. 10.2147/dddt.s99958 27103787 PMC4827895

[B63] Zhong Y. Shobo A. Hancock M. A. Multhaup G. (2019). Label-free distribution of anti-amyloid D-AIP in Drosophila melanogaster: prevention of Abeta42-induced toxicity without side effects in transgenic flies. *J. Neurochem.* 150 74–87. 10.1111/jnc.14720 31077378

