# Supplementary figures and images for: In vivo Evaluation of a Newly Synthesized Acetylcholinesterase Inhibitor in a Transgenic Drosophila Model of Alzheimer’s Disease
Source: Front Neurosci. 2021 Jun 30;15:691222. doi: 10.3389/fnins.2021.691222 (PMC8278008; doi:10.3389/fnins.2021.691222)

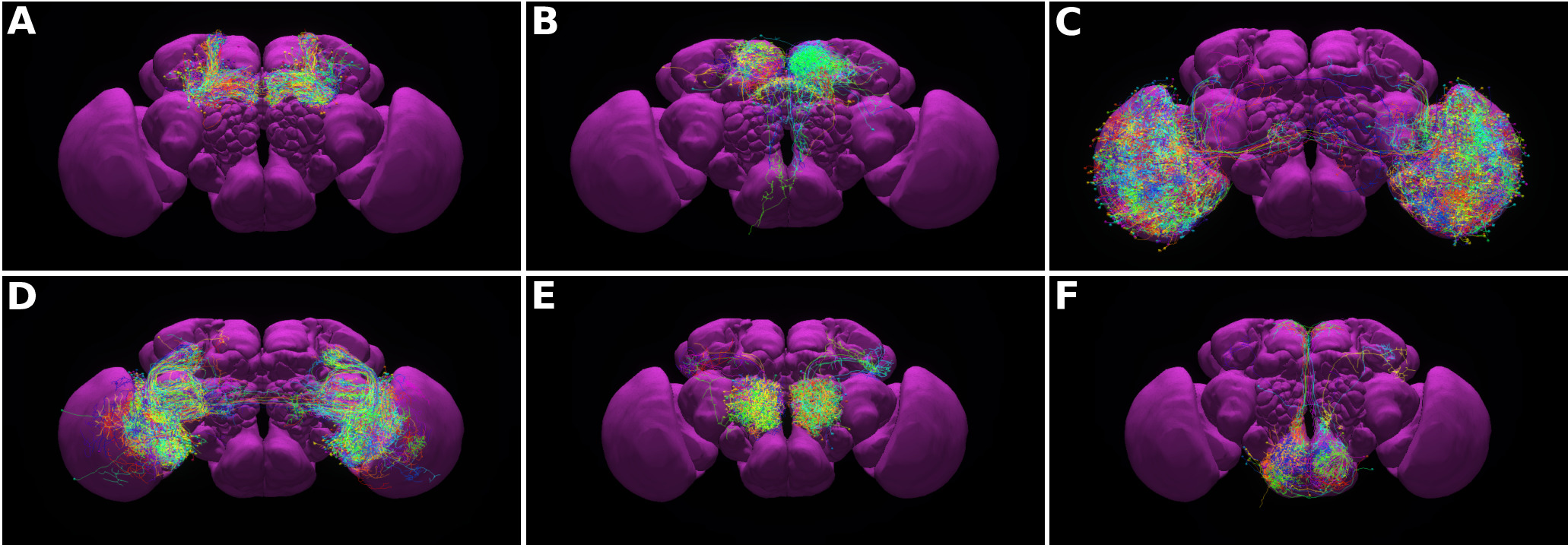

Supplement: Supplementary Figure 1 — Representative images of cholinergic neurons network in Drosophila Melanogaster brain obtained from the Virtual Fly Observatory. (A) Cholinergic neurons within the mushrooms bodies. (B) Cholinergic neurons within the fan-shaped bodies. (C) Cholinergic neurons within the medulla. (D) Cholinergic neurons within the optic lobes. (E) Cholinergic neurons within the antennal lobes. (F) Cholinergic neurons within the subesophageal ganglion. [file Image_1.JPEG]

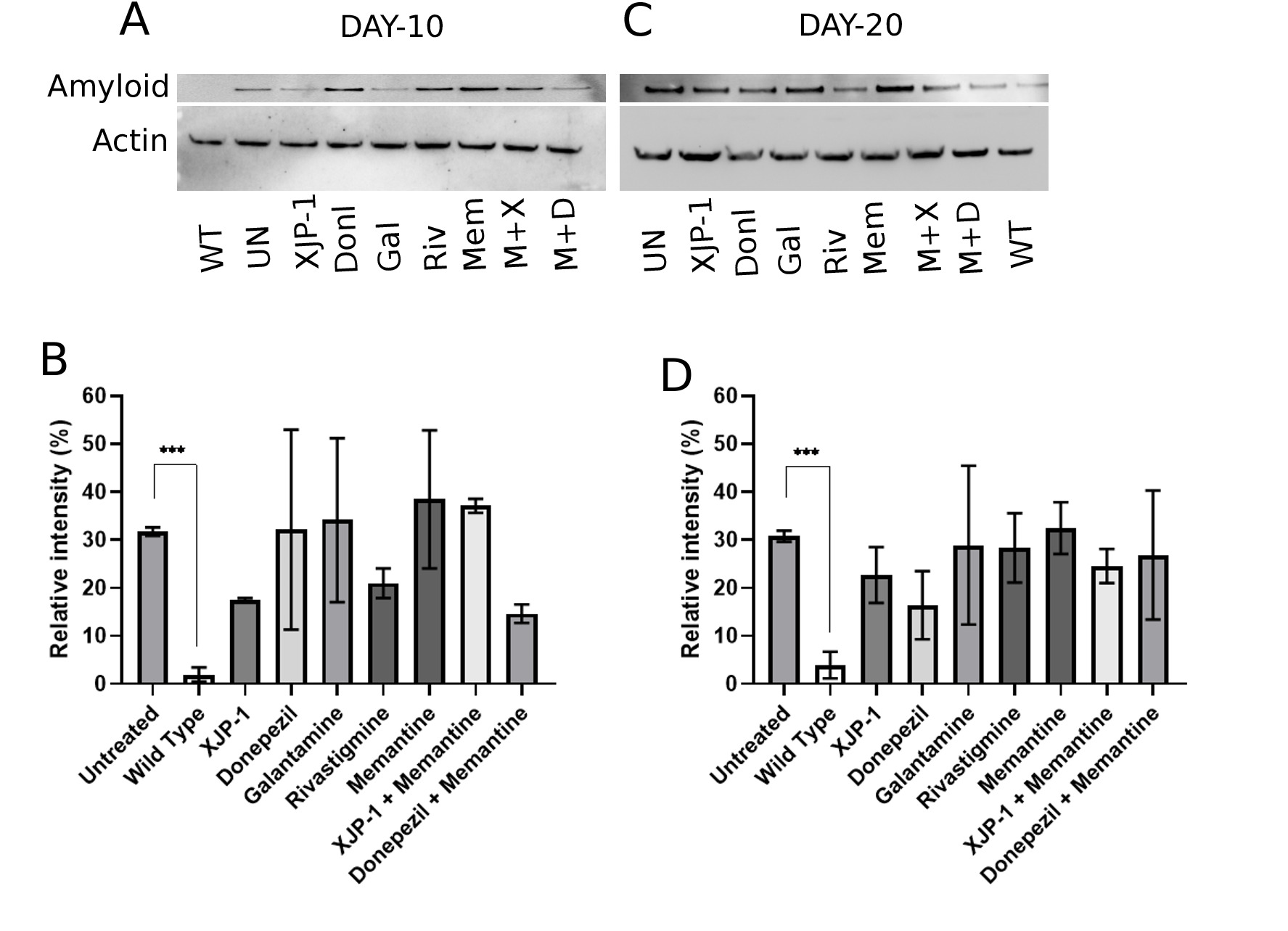

Supplement: Supplementary Figure 2 — Amyloid peptides quantity in Aβarc flies’ heads. (A) Representative images of WB membranes showing amyloid peptides quantity after 10 and 20 days of treatment. (B) AβArc peptide quantification by membrane image analysis. ANOVA test followed by Bonferroni’s post hoc was used to compare the differences between different groups. Data are presented as mean ± SEM, n = 3 (number of independent experiments with 50 fly heads per treatment group). P < 0.05 was considered as significant. ∗P < 0.05; ∗∗P < 0.001; ∗∗∗P < 0.0001. [file Image_2.JPEG]
